# Cost‐Effectiveness Analysis of Treatments for Children With Uncontrolled Asthma Symptoms Despite Inhaled Corticosteroids

**DOI:** 10.1002/ppul.71414

**Published:** 2025-12-15

**Authors:** Giovanna Culeddu, Sofia Cividini, Ian Sinha, Sarah Donegan, Katie Rose, Olivia Fulton, Stephen Turner, Catrin Tudur Smith, Dyfrig A. Hughes, EINSTEIN collaborative group

**Affiliations:** ^1^ Centre for Health Economics and Medicines Evaluation Bangor University Bangor Wales UK; ^2^ Institute of Population Health, Department of Health Data Science University of Liverpool Liverpool England UK; ^3^ Alder Hey Children's Foundation NHS Trust Liverpool England UK; ^4^ Patient Representative Liverpool England UK; ^5^ Women and Children Division NHS Grampian Aberdeen Scotland UK; ^6^ Institute of Applied Health Sciences University of Aberdeen Aberdeen Scotland UK

**Keywords:** asthma, cost‐effectiveness, economic evaluation, inhaled corticosteroids, pediatric

## Abstract

**Introduction:**

There is uncertainty about the cost‐effectiveness of treatment options for children and adolescents with uncontrolled asthma despite inhaled corticosteroid (ICS) treatment.

**Methods:**

A Markov state‐transition model was developed to simulate costs from the perspective of the National Health Service in the UK and health outcomes associated with low, medium and high dose ICS, ICS in combination with long‐acting β_2_‐adrenoceptor agonists (LABAs) or leukotriene receptor antagonists (LTRAs), and LTRA monotherapy. Healthcare resource use and health state utilities were identified from literature searches. Transition probabilities were derived from a systematic review and network meta‐analysis. Total costs and quality‐adjusted life years were computed, and incremental cost‐effectiveness ratios estimated over a 1‐year time horizon. Parameter, structural and probabilistic sensitivity analyses were performed.

**Results:**

Compared with low‐dose ICS, medium‐dose ICS and ICS + LABA had incremental cost‐effectiveness ratios of £255,555 and £304,956 per quality‐adjusted life year gained, respectively. High‐dose ICS, LTRA alone and in combination with ICS were dominated by alternatives which were less costly and associated with a greater number of quality‐adjusted life years. The incremental cost‐effectiveness ratio for medium‐dose ICS reduced to £14,797 per quality‐adjusted life year gained when the transition probabilities were increased to reflect a higher risk of asthma exacerbation. ICS + LABA became cost effective with a > 60% reduction in inhaler price.

**Conclusions:**

Treatment escalation beyond low‐dose ICS in children and adolescents with uncontrolled asthma may only be cost‐effective in the UK if the prices of alternatives reduce or treatment is targeted to those at higher risk of asthma exacerbations.

## 1 Introduction

Asthma is a prevalent, chronic respiratory condition affecting a significant number of children worldwide including over one million in the United Kingdom (UK) [1, 2]. Despite the widespread use of inhaled corticosteroids (ICS), a considerable proportion of children with asthma remain symptomatic, and experience exacerbations leading to impaired quality of life and increased healthcare utilization. In the UK, one child is admitted to hospital every 20 min due to an asthma exacerbation [3]. The financial impact of asthma is significant [4], totaling around £1.5 bn annually in direct National Health Service costs (all ages, 2019 prices) [5].

Following a diagnosis of asthma in children, a stepwise approach to treatment is recommended [6, 7]. Initial treatment typically involves the use of a low‐dose ICS to prevent symptoms, along with a short‐acting β_2_‐adrenoceptor agonist (SABA) for symptom relief. However, between 10% and 15% of children do not achieve adequate level of asthma control when using low‐dose ICS [8], requiring treatment that includes long‐acting β_2_‐adrenoceptor agonists (LABAs), leukotriene receptor antagonists (LTRAs) and/or increased dose of ICS [7].

The EstablishINg the best STEp‐up treatments for children with uncontrolled asthma despite INhaled corticosteroids (EINSTEIN) study [9, 10] involved a network meta‐analysis of individual participant data which demonstrated that medium‐dose ICS + LABA decreases the likelihood of exacerbation compared with low‐dose ICS, with an odds ratio (95% credibility interval) of 0.44 (0.19–0.90). Medium‐dose ICS + LABA increases FEV_1_ with a mean difference of 0.71 (0.35–1.06) compared with low‐dose ICS; 0.69 (0.33–1.05) compared with medium‐dose ICS; and 0.54 (0.24–0.81) compared to high‐dose ICS; whilst LTRA monotherapy (and alternative to low dose ICS in some guidelines) was the least effective option.

Given the large price differences among these medicines [11], the aim of this economic analysis was to assess the cost‐effectiveness of treatments in children who have poor asthma control, despite regular use of ICS. The analysis aims to assess the value for money of different treatment strategies and provide evidence to support clinicians, decision‐makers and policy‐makers in the management of pediatric asthma.

The primary objective was to estimate the incremental cost per quality‐adjusted life year (QALY) gained of alternatives to low‐dose ICS using a decision analytic model from the perspective of the National Health Services in the UK, and in accordance with the National Institute for Health and Care Excellence (NICE) methods [12].

## 2 Methods

### 2.1 Overview

To establish which treatments represent good value for money to the National Health Service in the UK, secondary data on clinical effectiveness, disease trajectory, healthcare resource use and health state utility (preference values that patients attach to their overall health status) were obtained from reviews of the literature. These were analyzed within a decision analytic model to simulate cohorts of patients (aged < 18 years who had uncontrolled asthma despite being prescribed low‐dose ICS) with respect to whether or not symptoms were controlled, and the occurrence of acute exacerbations or death. The model outputs include overall healthcare costs incurred and the number of QALYs experienced, on average, for each treatment. Comparison of costs and QALYs reveals whether treatments are cost‐effective—if the additional cost of achieving one additional QALY is less than £20,000, a treatment is generally considered to represent good value for money. The results are specific to the population modelled, and to the healthcare system in the UK.

### 2.2 Study Population and Treatments

The study considered a pediatric population aged < 18 years old of both sexes and of any ethnicity who were prescribed low‐dose ICS and had poor asthma control (i.e. uncontrolled asthma), consistent with the trials included in the network meta‐analysis [10]. The treatment comparators in the analysis represented current UK practice, and included: ICSs (beclometasone dipropionate, budesonide, ciclesonide, fluticasone furoate, fluticasone propionate, mometasone)—alone, or in combination with LABAs (formoterol, salmeterol, vilanterol) or LTRAs (montelukast, zafirlukast); and LTRA monotherapy.

For the economic analysis, the doses of ICSs were categorized as being low, medium and high, in accordance with the Global Initiative for Asthma (GINA) guideline [6]. Low‐ and medium‐dose ICS were represented by beclometasone dipropionate (100 µg and 200 µg/dose), high‐dose ICS by fluticasone propionate (250 µg/dose), low‐ and high‐dose ICS + LABA by fluticasone propionate (50 µg and 250 µg/dose) and salmeterol xinafoate (25 µg/dose), medium‐dose ICS + LABA by budesonide (200 µg/dose) and formoterol fumarate dihydrate (6 µg/dose), and ICS + LTRA by beclometasone dipropionate (200 µg/dose) and montelukast (10 mg). Each assumed access to SABA as required.

### 2.3 Economic Model

The structure and parameterization of the model were informed by existing economic evaluations of asthma treatments in children, identified via a literature search of five databases conducted between January 2000 and September 2020. The search strategy included the following medical subject headings and free text words which were combined with Boolean operators: “asthma” and (“children” or “pediatric” or “adolescents”) and (“decision analytic model” or “Markov model” or “decision tree”) and (“QALY” or “utilities”) and (“cost” or “costs”). Forty‐eight papers were identified in PubMed, 181 in Ovid Medline, 8 in Embase, 9 in the British Medical Journal database, and 5 in the Cochrane library. Nine economic models were identified and presented to clinical co‐investigators for comment on their face validity with respect to the clinical meaningfulness of health states and directionality of transition probabilities.

### 2.4 Markov Health States

**Figure 1:**
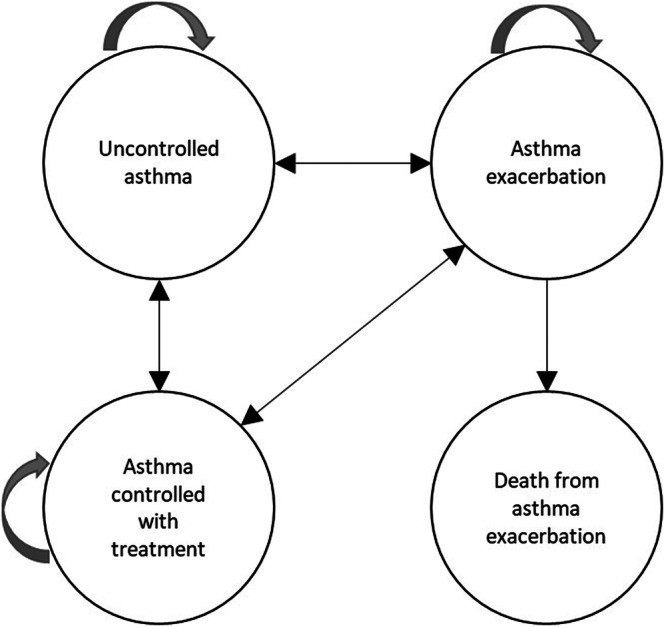
A schematic representation of the Markov economic model depicts the transitions between mutually exclusive health states for pediatric asthma patients. The arrows indicate the direction of movement among the health states.

The economic analysis comprised a Markov model to represent asthma according to four mutually exclusive health states. These were: “controlled asthma with treatment”, “uncontrolled asthma”, “asthma exacerbation”, and “death from asthma exacerbation”. Patients in the controlled asthma state were assumed to be managed in primary care and received ICS maintenance treatment. Those in the uncontrolled asthma state were defined by their need for emergency and secondary care consultations in addition to needing primary care clinic visits. Individuals in the asthma exacerbation state experienced severe symptom deterioration, requiring emergency department attendance, in‐patient care and out‐patient hospital follow‐up. The model included a death state for asthma‐related fatalities (Figure 1).

## 3 Transition Probabilities

The likelihood of patients receiving ICS moving between health states in a given cycle (their transition probabilities) was derived from published economic evaluations [13, 14], which were themselves based on data extracted from a systematic review that had identified five studies [15, 16, 17, 18, 19]. The generalizability of these studies to the population modelled in the present analysis was reviewed, and improved by assuming all patients entered the model in the “uncontrolled asthma” state, and that the transition probabilities for the base‐case analysis related to low‐dose ICS.

The effects of alternative treatments relative to low‐dose ICS were incorporated based on their relative risks of asthma control or exacerbation derived from a Bayesian fixed‐effect network meta‐analysis of 48 trials, which included patients aged 4 to < 18 years [10]. The annual probability of death from asthma exacerbation was calculated by adjusting mortality data for age [20], and converting to weekly probabilities, assuming a constant hazard function. The transition probability matrices are presented in the Supplementary Appendix: S1.

The Markov cycle length was defined as 1 week, and a half‐cycle correction was implemented. The model time horizon of analysis was 12 months.

## 4 Resource Utilization

A purposive review of the literature was conducted to identify resource use data to parameterize the economic model. This involved a citation search to find any economic evaluations linked to the 29 trials included in the network meta‐analysis and for which individual participant data were available [10], and a search of the PubMed, Ovid Medline, Embase, ECONLIT and Cochrane databases. The search was limited to studies published in the English language between January 2000 and October 2021 and filtered for a population aged < 18 years. Search terms relating to costs, asthma, pediatrics and economic evaluation were connected by Boolean operators.

There were no economic evaluations based on the trials used for the network meta‐analysis. The full texts of 85 studies identified in the review were examined, however, after careful evaluation and discussion with the clinical team, a decision was made to use data relating to the control group of the Reducing Asthma Attacks in Children using exhaled Nitric Oxide clinical trial (RAACENO, ISRCTN67875351) on the basis of greater generalizability to the United Kingdom, and detailed reporting of resource use and costs [21]. Twelve months resource utilization related to health services that were not associated to asthma exacerbation from the RAACENO trial was projected to the asthma‐controlled and uncontrolled health states within the EINSTEIN study. Uncontrolled health state costs included emergency and secondary care in addition to primary care costs. We assumed zero costs related to death. The total costs were recalibrated on a weekly basis to align with the EINSTEIN economic model's cycle length. Resource use related to asthma exacerbation episodes from the RAACENO trial was applied to the weekly cycle assigned to the asthma exacerbation in the EINSTEIN study.

Routine data on dispensed primary care prescriptions [22] do not distinguish between adult and pediatric prescriptions. Expert opinion was therefore sought from the clinical co‐investigators to identify the most commonly prescribed inhaler devices for children. It was assumed that 12 inhaler devices were issued per year. Quantities of resource use by health state are presented in Table 1.

**Table 1:** Resource Use (Numbers and Rates) by Health State, Per Year. Values are Means (standard deviations).

Items of resource use	Health state		
	Asthma controlled (per annum)	Asthma uncontrolled (per annum)	Asthma exacerbation (per exacerbation)
Primary and community care			
GP visit	0.75 (1.65)	0.75 (1.65)	0.79 (0.87)
Nurse consultation	0.15 (0.54)	0.15 (0.54)	0.11 (0.43)
NHS 24/111 calls	0.09 (0.51)	0.09 (0.51)	0.09 (0.42)
Out of hours GP consultations	0.04 (0.21)	0.04 (0.21)	0.05 (0.23)
Walk‐in consultations	0.05 (0.27)	0.05 (0.27)	0.05 (0.24)
Pharmacist consultations	0.01 (0.10)	0.01 (0.10)	0
Secondary and emergency care			
Emergency department visits	0	0.09 (0.32)	0.2 (0.45)
Hospital inpatient stays	0	0.02 (0.15)	0.14 (0.38)
Hospital outpatient clinic attendance	0	0.25 (0.92)	0.03 (0.17
Day case	0	0	0.00 (0.06)
Bronchoscopy	0	0.01 (0.10)	0
Ambulance	0	0.01 (0.11)	0.03 (0.20)
Other (physiotherapist/psychologist/speech and language therapist)	0	0.01 (0.14)	0
Medicines			
Prescription items for treatments (numinhalers or pack of 28 tablets)^a^	12 (2)	12 (2)	0.14 (0.1)
SABA reliever inhaler	1 (0.20)	1 (0.20)	0.02 (0.20)

^a^ Assumed values of standard deviation.

## 5 Unit Costs

Unit costs were assigned to each item of resource use, based on the latest available at the time of analysis (2019/20) and expressed in pounds sterling (£GBP). Healthcare Resource Group (HRG) codes were identified for emergency department attendances, hospital outpatient clinic visits, in‐patient stays and day case consultations and costed using the National Health Service Schedule of Reference Costs 2018–19 (Table 2) [23]. Unit costs of consultations with general practitioners were sourced from the Compendium of Unit Costs of Health and Social Care 2020 [24]. Costs were inflated, where applicable, using the National Health Service cost inflation index [24].

The asthma medicines used in the analysis are listed in Table 2 and their unit costs were taken as the Drug Tariff price from the British National Formulary [25]. The weekly cost of inhaler devices was calculated based on dividing the daily cost of the prescribed dose by the number of actuations per inhaler device, then multiplying by seven.

**Table 2:** Unit Costs of Resource Items.

Items of resource use	Cost per episode/item (£)	Reference
Primary and community care		
GP visit	39.65	[24]
Nurse consultation	24.00	[24]
NHS 24/111 call	12.96	[37]
Out of hours GP (Weighted average of T03A and T03NA ‐ excluding emergency dental)	74.02	[23]
Walk‐in consultation (Weighted average of T04A and T04NA ‐ excluding emergency dental)	45.71	[23, 24]
Secondary and emergency care		
ED (HRG code VB09Z Emergency medicine, category 1 investigation with category 1–2 treatment (type 1 non‐admitted))	133.00	[23]
ED (HRG codes VB06Z and VB04Z weighted average by severity of admission)	264.00	[23]
Hospital short stay (admitted for ≤ 1 night) (Weighted average of PD12 Paediatric, Asthma or Wheezing)	594.00	[23]
Hospital long stay (admitted for ≤ 4 days and > 1 night (Weighted average of PD12 Paediatric, Asthma or Wheezing)	1913.00	[23, 24]
Excess bed‐days (Weighted average of PD12 Paediatric, Asthma or Wheezing)	575.00	[23, 24]
Hospital day case (Weighted average of PD12 Paediatric, Asthma or Wheezing)	394.00	[23]
Bronchoscopy (DZ69B Diagnostic Bronchoscopy, 18 years and under, combined day case/ordinary elective spell tariff)	952.00	[36]
Ambulance (see and treat)	209.00	[36]
Ambulance (see and convey)	257.00	[36]
Clinical psychologist	54.00	[24]
Physiotherapist	57.00	[24]
Speech and language therapist	34.00	[24]
Medicines		
Low‐dose ICS: beclometasone dipropionate (Clenil Modulite 100 µg/dose inhaler)	7.42	[25]
Medium‐dose ICS: beclometasone dipropionate (Clenil Modulite 200 µg/dose inhaler)	16.17	[25]
Medium‐dose ICS + LABA: budesonide and formoterol fumarate dihydrate (Symbicort 200/6 Turbohaler)^a^	28.00	[25]
ICS + LTRA: beclometasone dipropionate (Clenil Modulite 200 µg/dose inhaler) and montelukast 10 mg tablets	17.87	[25]
High‐dose ICS: Fluticasone propionate (Flixotide 250 µg/dose Evohaler)	36.14	[25]
Low‐dose ICS + LABA: Fluticasone propionate 50 µg/dose and salmeterol xinafoate 25 µg/dose (Seretide 50 Evohaler)	17.46	[25]
High‐dose ICS + LABA: Fluticasone propionate 250 µg/dose and salmeterol xinafoate 25 µg/dose (AirFluSal inhaler)	29.32	[25]
SABA: salbutamol 100 µg/dose (Airomir)	1.50	[25]

^a^ This cost was assumed for ICS + LABA.

## 6 Health State Utilities

To calculate the number of QALYs experienced by patients, health utilities in pediatric asthma were sourced by updating a previously published systematic review of literature [26] for the period between July 2014 and October 2021. In addition to the 927 studies identified previously, the search returned 2833 publications. The full texts of 86 articles were reviewed. Studies were selected which reported utility scores using the EuroQol EQ‐5D generic, preference‐based measure, in line with NICE's preferred multi‐attribute health utility instrument [12].

For the “controlled asthma” state, a utility weight of 0.96 was applied, based on the standard care group of a trial involving a population of adults and children with mild to moderate asthma [27], and in which the United Kingdom valuation set of EQ‐5D‐3L was used [28]. For the “uncontrolled asthma” and “asthma exacerbation” health states, disutilities of 0.10 and 0.20 were applied (as deductions from 0.96), respectively. These values were sourced from a population of adult patients with moderate or severe asthma who had experienced exacerbations [29]. These utility values align with previous economic evaluations [30, 31], and are consistent with the NICE technology appraisal of inhaled corticosteroids for the treatment of chronic asthma in children under the age of 12 [32].

## 7 Incremental Analysis

In the primary cost‐utility analysis, and in accordance with standard methods of health economic evaluation, treatments were ranked in decreasing order of QALYs and inspected for dominance. Those that were dominated (more expensive and less effective than one or more alternative) were removed.

The incremental cost per QALY gained (ICER) for the remaining treatments, was calculated according to:

ICER = ΔCosts/ΔQALY

where, ΔCosts is the difference in mean total costs, and ΔQALY is the difference in mean QALYs, between a treatment and the next best alternative. Extendedly dominated treatments (those with an ICER higher than that of the next, more effective, alternative) were removed. ICERs were compared with the threshold for cost‐effectiveness operating in the UK, which is £20,000 per QALY gained [12].

Secondary analyses of cost‐effectiveness considered the incremental costs per exacerbation‐free day, and costs per additional day of controlled asthma.

## 8 Analysis of Parameter Uncertainty

One‐way sensitivity analyses were conducted to assess the stability of the ICER to different assumptions or ranges of parameter estimates. The ranges were based on confidence intervals or assumed limits. These following variables were considered:

i. varying point estimate utility values by ±0.05, and between the lower and upper bounds of their 95% confidence intervals
ii. reducing the cost of branded inhaler products by 50% (plausible representation of the price of generic inhalers); and in a threshold analysis, by an amount sufficient to achieve cost‐effectiveness
iii. increasing the transition probability of “asthma exacerbation” by 50% concurrently for all treatments, while compensating with a reduction in transition probabilities for “asthma controlled” (assumed plausible range).

### 8.1 Analysis of Structural Uncertainty

A number of analyses were undertaken to assess the impact on the base‐case ICER of uncertainties that arise from the inherent limitations and simplifications of the economic model. These were:

i. applying random effects Bayesian probabilities for the calculation of relative risks of transition probabilities relating to “controlled asthma” and “asthma exacerbation”.
ii. assuming that the transition probabilities from Rodriguez‐Martinez et. al. (2015) [13] applied to medium‐dose (rather than low‐dose) ICS. This was based on the observation that some studies included doses in the range as defined by the GINA guideline [6].
iii. applying random effect Bayesian probabilities for the calculation of relative risks of transition probabilities relating to “controlled asthma” and “asthma exacerbation”, and with medium‐dose ICS as a reference.
iv. separating the dose of ICS into low, medium and high when combined with LABA. This distinguished from the base‐case analysis with respect to relative risks (and hence transition probabilities), and cost (individually priced inhalers instead of assuming the cost of medium‐dose ICS when combined with LABA).

### 8.2 Probabilistic Sensitivity Analysis

A probabilistic sensitivity analysis was undertaken using a Monte Carlo simulation with 10,000 replicates to evaluate the joint uncertainty in costs and QALYs. Gamma distributions were assumed for transformed (1‐utility) scores with fixed standard deviations of 0.2 [33]. Items of resource use were assumed to be log‐normally distributed and drawn samples were multiplied by their respective (fixed) average unit costs. Beta distributions were fitted to fixed and random effect Bayesian probabilities. The parameters of each distribution is presented in Supplementary Appendix: S1.

A cost‐effectiveness acceptability curve was generated to depict the probability of each intervention being cost‐effective at different willingness‐to‐pay thresholds [34].

### 8.3 Patient and Public Involvement

The views of two parents of children with asthma and one adolescent patient were sought in the development of the study; and a patient and public involvement representative (OF) has been involved as a research team member from the planning stage through to completion. They advised on the importance of the research, selection of outcome measures, and the interpretation of the findings.

### 8.4 Reporting

The economic analysis is reported in accordance with the Consolidated Health Economic Evaluation Reporting Standards [35].

### 8.5 Ethical Approval

The study used anonymized data and data available in the public domain hence ethical approval was not required. The University of Liverpool Research Ethics Committee confirmed this before the start of the project.

### 8.6 Informed Consent

Consent was not sought as no participants were recruited to this study. The research involved the secondary analysis of existing, published, anonymized data.

## 9 Results

Patients resided mainly in the “controlled asthma” state, ranging from 80.5% of the time with LTRA monotherapy to 92.0% with ICS + LABA. Accordingly, the majority of costs and QALYs were accrued in this health state, with the exception of LTRA monotherapy for which the majority of costs were associated with the asthma exacerbation health state (Supplementary Appendix: S1).

In the base‐case analysis, and over the 52 weeks of the model time horizon, LTRA monotherapy was associated with the highest cost (£670), and low‐dose ICS the lowest (£284). ICS + LABA provided the highest number of QALYs with 0.9512, and LTRA monotherapy the least (0.9366 QALYs) (Table 3).

**Table 3:** Results of the Incremental Analyses.

Outcome	Total Cost (£)	Outcome	Incremental Cost (£)	Incremental benefit	ICER (£/unit of benefit gained)
Treatment					
Outcome = QALYs					
ICS + LABA	501	0.9512	124	0.0004	304,956
Medium‐dose ICS	377	0.9508	93	0.0004	255,555
Low‐dose ICS	284	0.9504	—	—	0
High‐dose ICS	470	0.9503			Dominated
ICS + LTRA	596	0.9495			Dominated
LTRA	670	0.9366			Dominated
Outcome = Days of controlled asthma					
ICS + LABA	501	335.6	124	1.0	118
Medium‐dose ICS	377	334.6	93	1.1	88
Low‐dose ICS	284	333.5	—	—	—
LTRA	670	293.8			Dominated
High‐dose ICS	470	332.2			Dominated
ICS + LTRA	377	331.5			Dominated
Outcome = Exacerbation‐free days					
Medium‐dose ICS	377	361.8	93	0.3	341
Low‐dose ICS	284	361.5	—	—	—
High‐dose ICS	470	362.4			Extendedly dominated
ICS + LABA	501	362.2			Dominated
ICS + LTRA	377	360.0			Dominated
LTRA	670	350.8			Dominated

### 9.1 Incremental Analysis

Analyzed incrementally, low‐dose ICS was the cost‐effective choice, associated with a cost of £284 and 0.9504 QALYs (Table 3). Medium‐dose ICS and ICS + LABA were not cost‐effective options, with ICERs of £255,555 and £304,956 per QALY gained, respectively. High‐dose ICS, ICS + LTRA and LTRA monotherapy were dominated by alternatives which were less costly and associated with a greater number of QALYs.

ICS + LABA was associated with the highest number of days per year of controlled asthma (335.6 days) and provided, on average, a further 1.0 day of controlled asthma over medium‐dose ICS alone, at an incremental cost of £124. Medium‐dose ICS provided an extra day of asthma control over low‐dose ICS at an incremental cost of £88 (Table 3). All other treatments were dominated. The incremental cost per exacerbation‐free day was £341 for medium‐dose ICS versus low‐dose ICS, while the other treatments were either dominated or extendedly dominated. LTRA monotherapy resulted in significantly more days per year spent in the exacerbation health state, being five and four times higher than those modelled with ICS + LABA and low‐dose ICS, respectively (Table 3).

### 9.2 Sensitivity Analysis

When the costs of branded inhalers were individually discounted by 50%, the ICER for ICS + LABA reduced considerably, but it remained non‐cost‐effective (Supplementary Appendix: S1). None of the other treatment options were cost‐effective in this sensitivity analysis, with high‐dose ICS, ICS + LTRA and LTRA monotherapy being consistently dominated. The threshold analysis indicated that the price of medium‐dose ICS + LABA would need to decrease by 60.4% (from £16.17 to £9.06 per inhaler) to become cost‐effective.

Medium‐dose ICS became cost‐effective at £14,797 per QALY gained when the transition probabilities associated with “asthma exacerbation” and “asthma controlled” were varied by ±50% concurrently for all treatments (Supplementary Appendix: S1).

The results of one‐way sensitivity analyses applied to utilities associated with the “uncontrolled asthma” and “asthma exacerbation” health states indicated no impact on the rank‐ordering of treatments by cost‐effectiveness (Supplementary Appendix: S1).

### 9.3 Analysis of Structural Uncertainty

Across all structural uncertainty analyses, none of the treatment options became cost‐effective. When LABA was added to various strengths of ICS, medium‐dose ICS + LABA generated an additional cost of £113 and 0.0005 more QALYs than medium‐dose ICS alone. High‐dose ICS (alone and + LABA), low‐dose ICS + LABA, ICS + LTRA, and LTRA monotherapy, were all dominated. ICERs were comparable to the base‐case analysis when using relative risks from the Bayesian random effects network meta‐analysis. Treatment with medium‐dose ICS, both as a standalone and when combined with LABA, was typically associated to improved QALYs, while low‐dose ICS was consistently the least costly non‐dominated treatment.

### 9.4 Probabilistic Sensitivity Analysis

**Figure 2:**
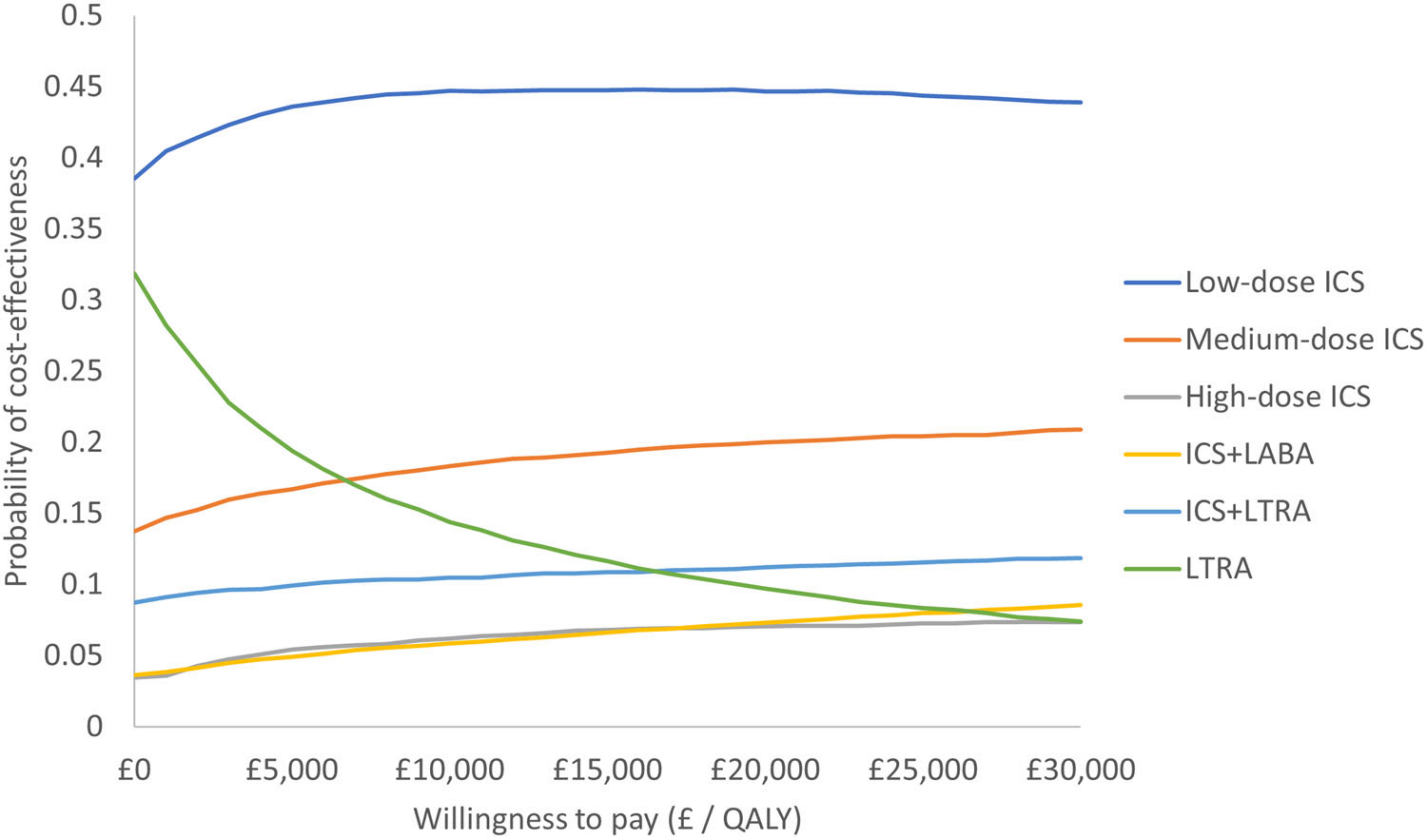
Cost‐effectiveness acceptability curves depicting the probability of each treatment's cost‐effectiveness for a range of willingness to pay values. [Color figure can be viewed at wileyonlinelibrary.com]

The results of the probabilistic sensitivity analyses were consistent with the deterministic analyses (Table 4). The cost‐effectiveness acceptability curve indicated low‐dose ICS to have the highest probability of being the most cost‐effective option at 0.45 for a threshold of £20,000 per QALY (Figure 2). At the £20,000 per QALY threshold, the cost‐effectiveness probabilities for ICS + LABA and medium‐dose ICS were 0.07 and 0.20, respectively.

**Table 4:** Results of the Probabilistic Sensitivity Analysis.

Treatment	Total cost (£) (95% central range)	Total QALY (95% central range)	Probability of cost‐effectiveness at:	
			£20,000 per QALY	£30,000 per QALY
Low‐dose ICS	281 (63, 974)	0.9505 (0.5800, 0.9991)	0.45	0.44
Medium‐dose ICS	381 (114, 1108)	0.9509 (0.5791, 0.9992)	0.20	0.21
ICS + LTRA	476 (127, 1519)	0.9491 (0.5796, 0.9990)	0.11	0.12
LTRA	678 (40, 3864)	0.9333 (0.6073, 0.9980)	0.10	0.07
ICS + LABA	498 (175, 1223)	0.9513 (0.5794, 0.9993)	0.07	0.09
High‐dose ICS	812 (158, 2913)	0.8656 (0.3449, 0.9978)	0.07	0.07

## 10 Discussion

The cost‐utility analysis indicated that step‐up treatment options for children and adolescents who are not effectively controlled by low‐dose ICS (plus SABA as required) are not cost‐effective at current prices. ICERs for medium‐dose ICS and ICS + LABA (each with SABA as required) far exceed willingness to pay thresholds operating in the United Kingdom [12]. High‐dose ICS, ICS + LTRA and LTRA monotherapy were dominated, that is, they were modelled to be more costly and associated with fewer QALYs than alternatives.

These results were mostly stable to sensitivity analyses that considered parameter and structural uncertainty. However, in scenarios where the likelihood of exacerbations increased, medium‐dose ICS became the cost‐effective option at the £20,000 per QALY threshold. Reducing the cost of treatments did not significantly alter the cost‐effectiveness of the assessed treatments. Notably, however, reducing the price of medium‐dose ICS + LABA inhalers by > 60% led to this combination becoming cost‐effective. The sensitivity analyses, therefore, suggest that asthma severity and the price of inhalers are influential determinants of the cost‐effectiveness of treatments.

Differences in QALYs among the evaluated treatments reflected the time patients resided in each of the health states and were consistent with the ordering of treatments based on asthma control or exacerbation rates obtained from the fixed‐effect network meta‐analysis [10]. In other words, treatments that demonstrated higher effectiveness in controlling asthma or preventing exacerbations also yielded higher total QALYs, corresponding to better overall health‐related quality of life for patients. Our analysis, however, did not adjust for treatment effect modifiers, such as variable adherence, that may differ between the rarified context of clinical trials and routine practice [38].

There were many limitations to this study that necessitated several assumptions to construct the model in the absence of a direct comparison of treatment options. It was assumed that health states and transition probabilities for low‐dose ICS derived from a previous economic model [13, 14] applied to the study population. While the trials that informed the previous model were of children prescribed continuous dosing of ICS, the aim of these trials was primarily to assess as required ICS treatment and, as such, patients might have been adequately managed with low‐dose ICS plus a bronchodilator. The clinical trials recruited children described as being newly‐diagnosed [15], symptomatic with frequent wheezing episodes [16], with mildly persistent asthma [18], and with recurrent wheezing episodes, and at least one exacerbation in the previous year but a low degree of impairment [18]. A sensitivity analysis in which the risk of exacerbation was increased to reflect increased disease severity, resulted in medium‐dose ICS becoming cost‐effective. A further limitation related to not taking into consideration the relative safety of the treatment options. High‐dose ICS, for instance, is associated with adrenal and growth suppression [39] and has implications for costs and health‐related quality of life.

An issue pertinent to our analysis was the decision to include low‐dose ICS as a comparator. Arguably, if asthma is uncontrolled, the decision problem should only consider alternative options, and exclude low‐dose ICS as a valid comparator. Based on this, medium‐dose ICS would be the optimal choice, as ICS + LABA remains non‐cost‐effective at £304,956 per QALY gained. The recent British Thoracic Society, NICE, Scottish Intercollegiate Guidelines Network joint guideline on the diagnosis, monitoring and management of chronic asthma [7] included an economic analysis of treatment escalation in children. As comparators, it considered low‐dose ICS + LABA with and without SABA as required, and moderate‐dose ICS with SABA as required. Consistent with our findings, their results indicated that low‐ or moderate‐dose ICS + LABA were not cost‐effective [40].

Despite the aim of the research being to identify the optimal step‐up treatment for children and adolescents who remain uncontrolled despite low‐dose ICS, the economic analysis indicated that other treatment options do not currently offer good value for money. However, should the prices of alternatives reduce significantly, they might become economically viable options. Medium‐dose ICS with or without LABA were not cost‐effective but were associated with more modelled quality‐adjusted life years than the other comparators considered. Future research should be directed towards comparing treatments directly to establish comparative clinical effectiveness and enable more accurate modelling of cost‐effectiveness.

## Author Contributions

**Giovanna Culeddu:** methodology, investigation, formal analysis, writing – original draft. **Sofia Cividini:** investigation, writing – review and editing, formal analysis. **Ian Sinha:** conceptualization, funding acquisition, writing – review and editing, project administration. **Sarah Donegan:** funding acquisition, writing – review and editing. **Katie Rose:** writing – review and editing, investigation, formal analysis. **Olivia Fulton:** funding acquisition, writing – review and editing. **Stephen Turner:** funding acquisition, writing – review and editing, investigation. **Catrin Tudur Smith:** conceptualization, funding acquisition, writing – review and editing; project administration, investigation. **Dyfrig A. Hughes:** conceptualization, funding acquisition, investigation, methodology, writing – review and editing, writing – original draft, project administration.

## Collaborators

Members of the EINSTEIN collaborative group are listed in the Supplementary Appendix.

## Conflicts of Interest

The authors declare no conflicts of interest.

## Supporting information

Supplementary appendix EINSTEIN CEA PP.

## Data Availability

Data sharing is not applicable to this article as the study used secondary data available from published sources. The study used secondary data available from published sources.

## References

[1] The Global Asthma Report 2022 . International Journal of Tuberculosis and Lung Disease 26, suppl. 1 (2022): 1–104. 10.5588/ijtld.22.1010 36303302

[2] M. I. Asher , C. E. Rutter , K. Bissell , et al., Global Asthma Network Phase I Study Group , “Worldwide Trends in the Burden of Asthma Symptoms in School‐Aged Children: Global Asthma Network Phase I Cross‐Sectional Study,” Lancet 398, no. 10311 (2021): 1569–1580. 34755626 10.1016/S0140-6736(21)01450-1 PMC8573635

[3] NHS England , NHS Warning to Parents as Asthma Season Hits (NHS England, 2019), https://www.england.nhs.uk/2019/09/nhs-warning-to-parents-as-asthma-season-hits/.

[4] M. Mukherjee , A. Stoddart , R. P. Gupta , et al., “The Epidemiology, Healthcare and Societal Burden and Costs of Asthma in the UK and Its Member Nations: Analyses of Standalone and Linked National Databases,” BMC Medicine 14, no. 1 (2016): 113. 27568881 10.1186/s12916-016-0657-8 PMC5002970

[5] Asthma and Lung UK , *Investing in Breath: Reducing the Economic Cost of Lung Conditions Through Increased Research and Innovation*, PWC Report (2023), https://www.asthmaandlung.org.uk/investinginbreath-reducingeconomiccostoflungconditions-autumn-2023.

[6] GINA Science Committee , Global Strategy for Asthma Management and Prevention (Global Initiative for Asthma (GINA), 2019), https://ginasthma.org/reports/2019-gina-report-global-strategy-for-asthma-management-and-prevention/.

[7] National Institute for Health and Care Excellence. Asthma: Diagnosis, Monitoring and Chronic Asthma Management (BTS, NICE, SIGN) NG245. November 2024, https://www.nice.org.uk/guidance/ng245. 39937939

[8] S. Turner , M. Thomas , J. von Ziegenweidt , and D. Price , “Prescribing Trends in Asthma: A Longitudinal Observational Study,” Archives of Disease in Childhood 94, no. 1 (2009): 16–22. 18701558 10.1136/adc.2008.140681

[9] S. Cividini , I. Sinha , S. Donegan , et al., “Establishing the Best Step‐Up Treatments for Children With Uncontrolled Asthma Despite INHaled Corticosteroids (EINSTEIN): Protocol for a Systematic Review, Network Meta‐Analysis and Cost‐Effectiveness Analysis Using Individual Participant Data (IPD),” BMJ Open 11, no. 2 (2021): e040528. 10.1136/bmjopen-2020-040528 PMC7925932 33550231

[10] S. Cividini , I. Sinha , S. Donegan , et al., “Best Step‐Up Treatments for Children With Uncontrolled Asthma: A Systematic Review and Network Meta‐Analysis of Individual Participant Data,” European Respiratory Journal 62, no. 6 (2023): 2301011. 37945034 10.1183/13993003.01011-2023 PMC10752294

[11] S. O'Neill , J. Sweeney , C. C. Patterson , et al., “The Cost of Treating Severe Refractory Asthma in the UK: An Economic Analysis From the British Thoracic Society Difficult Asthma Registry,” Thorax 70, no. 4 (2015): 376–378. 24917087 10.1136/thoraxjnl-2013-204114

[12] National Institute for Health and Care Excellence, NICE Health Technology Evaluations: The Manual, 2022, https://www.nice.org.uk/process/pmg36/.

[13] C. E. Rodriguez‐Martinez , G. Nino , and J. A. Castro‐Rodriguez , “Cost‐Utility Analysis of Daily Versus Intermittent Inhaled Corticosteroids in Mild‐Persistent Asthma,” Pediatric Pulmonology 50, no. 8 (2015): 735–746. 24965279 10.1002/ppul.23073 PMC5538803

[14] C. E. Rodriguez‐Martinez , M. P. Sossa‐Briceño , and J. A. Castro‐Rodriguez , “Cost‐Utility Analysis of Once‐Daily Versus Twice‐Daily Inhaled Corticosteroid Dosing for Maintenance Treatment of Asthma in Pediatric Patients,” Journal of Asthma 53, no. 5 (2016): 538–545. 10.3109/02770903.2015.1116087 26786524

[15] M. Turpeinen , K. Nikander , A. S. Pelkonen , et al., “Daily Versus As‐Needed Inhaled Corticosteroid for Mild Persistent Asthma (The Helsinki Early Intervention Childhood Asthma Study),” Archives of Disease in Childhood 93, no. 8 (2008): 654–659. 17634183 10.1136/adc.2007.116632 PMC2532957

[16] A. Papi , G. Nicolini , E. Baraldi , et al., “Regularvsprn Nebulized Treatment in Wheeze Preschool Children,” Allergy 64, no. 10 (2009): 1463–1471. 19772514 10.1111/j.1398-9995.2009.02134.x

[17] F. D. Martinez , V. M. Chinchilli , W. J. Morgan , et al., “Use of Beclomethasone Dipropionate as Rescue Treatment for Children With Mild Persistent Asthma (TREXA): A Randomised, Double‐Blind, Placebo‐Controlled Trial,” The Lancet 377, no. 9766 (2011): 650–657. 10.1016/S0140-6736(10)62145-9 PMC4852146 21324520

[18] R. S. Zeiger , D. Mauger , L. B. Bacharier , et al., “Daily or Intermittent Budesonide in Preschool Children With Recurrent Wheezing,” New England Journal of Medicine 365, no. 21 (2011): 1990–2001. 22111718 10.1056/NEJMoa1104647 PMC3247621

[19] C. E. Rodríguez‐Martínez , M. P. Sossa‐Briceño , and J. A. Castro‐Rodriguez , “Cost‐Utility Analysis of the Inhaled Steroids Available in a Developing Country for the Management of Pediatric Patients With Persistent Asthma,” Journal of Asthma 50, no. 4 (2013): 410–418. 10.3109/02770903.2013.767909 23356720

[20] C. I. Bloom , F. Nissen , I. J. Douglas , L. Smeeth , P. Cullinan , and J. K. Quint , “Exacerbation Risk and Characterisation of the UK's Asthma Population From Infants to Old Age,” Thorax 73, no. 4 (2018): 313–320. 29074814 10.1136/thoraxjnl-2017-210650

[21] S. Turner , S. Cotton , J. Wood , et al., “Treatment Guided by Fractional Exhaled Nitric Oxide In Addition to Standard Care in 6‐ to 15‐year‐olds With Asthma: The Raaceno RCT,” Efficacy and Mechanism Evaluation 9, no. 4 (2022), 10.3310/AWOI5587. 35679443

[22] A. Morgan , E. Maslova , C. Kallis , et al., “Short‐Acting β2‐agonists and Exacerbations in Children With Asthma in England: SABINA Junior,” ERJ Open Research 9, no. 2 (2023): 00571‐2022. 37101737 10.1183/23120541.00571-2022 PMC10123517

[23] NHS England , 2018/19 National Cost Collection Data Publication (NHS England, 2021), https://www.england.nhs.uk/publication/2018-19-national-cost-collection-data-publication/.

[24] L. Curtis , Unit Costs of Health and Social Care 2020, Personal Social Services Research Unit, University of Kent. https://www.pssru.ac.uk/project-pages/unit-costs/unit-costs-2020/.

[25] Joint Formulary Committee , British National Formulary (BMJ Group and Pharmaceutical Press, 2021).

[26] W. S. Kua and S. Davis , “Systematic Review of Health State Utilities in Children With Asthma,” Health Economics & Decision Science (HEDS) Discussion Paper Series (School of Health and Related Research, University of Sheffield, 2016), https://eprints.whiterose.ac.uk/102188/1/16.12.pdf.

[27] D. C. Willems , M. A. Joore , J. J. Hendriks , E. F. Wouters , and J. L. Severens , “Cost‐Effectiveness of a Nurse‐Led Telemonitoring Intervention Based on Peak Expiratory Flow Measurements in Asthmatics: Results of a Randomised Controlled Trial,” Cost Effectiveness and Resource Allocation 5 (2007): 10. 17662113 10.1186/1478-7547-5-10 PMC2000864

[28] MVH Group , Final Report on the Modelling of Valuation Tariffs, Centre for Health Economics, (University of York, 1995), https://www.york.ac.uk/media/che/documents/reports/MVH%20Final%20Report.pdf.

[29] A. Lloyd , D. Price , and R. Brown , “The Impact of Asthma Exacerbations on Health‐Related Quality of Life in Moderate to Severe Asthma Patients in the UK,” Primary Care Respiratory Journal 16, no. 1 (2007): 22–27. 10.3132/pcrj.2007.00002 PMC6634181 17297523

[30] S. A. Julious , M. J. Horspool , S. Davis , et al., “Open‐Label, Cluster Randomised Controlled Trial and Economic Evaluation of a Brief Letter From a GP on Unscheduled Medical Contacts Associated With the Start of the School Year: The PLEASANT Trial,” BMJ Open 8, no. 4 (2018): e017367. 10.1136/bmjopen-2017-017367 PMC5914776 29678962

[31] J. M. FitzGerald , S. Arnetorp , C. Smare , et al., “The Cost‐Effectiveness of As‐Needed Budesonide/Formoterol Versus Low‐Dose Inhaled Corticosteroid Maintenance Therapy in Patients With Mild Asthma in the UK,” Respiratory Medicine 171 (2020): 106079. 32917353 10.1016/j.rmed.2020.106079

[32] National Institute for Health and Care Excellence , Inhaled Corticosteroids for the Treatment of Chronic Asthma in Children Under the Age of 12 years (TA131) (2007), https://www.nice.org.uk/guidance/ta131.

[33] E. D. Bateman , J. Bousquet , W. W. Busse , et al., “Stability of Asthma Control With Regular Treatment: An Analysis of the Gaining Optimal Asthma Control (GOAL) Study,” Allergy 63, no. 7 (2008): 932–938. 18588561 10.1111/j.1398-9995.2008.01724.x

[34] E. Fenwick , K. Claxton , and M. Sculpher , “Representing Uncertainty: The Role of Cost‐Effectiveness Acceptability Curves,” Health Economics 10, no. 8 (2001): 779–787. 11747057 10.1002/hec.635

[35] D. Husereau , M. Drummond , F. Augustovski , et al.; CHEERS, “Consolidated Health Economic Evaluation Reporting Standards 2022 (CHEERS 2022) Statement: Updated Reporting Guidance for Health Economic Evaluations,” Value in Health 25, no. 1 (2022): 3–9. 35031096 10.1016/j.jval.2021.11.1351

[36] NHS England, Health Trust Reference Costs: 2017–18 (NHS England, 2018), https://www.data.gov.uk/dataset/71367291-5636-460f-a8fe-374780a16a8e/health-trust-reference-costs-2017-18.

[37] C. Pope , J. Turnbull , J. Jones , J. Prichard , A. Rowsell , and S. Halford , “Has the NHS 111 Urgent Care Telephone Service Been a Success? Case Study and Secondary Data Analysis in England,” BMJ Open 7, no. 5 (2017): e014815. 10.1136/bmjopen-2016-014815 PMC5623427 28576895

[38] B. Vrijens , A. L. Dima , E. Van Ganse , et al., “What We Mean When We Talk About Adherence in Respiratory Medicine,” The Journal of Allergy and Clinical Immunology: In Practice 4, no. 5 (2016): 802–812. 27587314 10.1016/j.jaip.2016.05.019

[39] J. S. Leung , D. W. Johnson , A. J. Sperou , et al., “A Systematic Review of Adverse Drug Events Associated With Administration of Common Asthma Medications in Children,” PLoS One 12, no. 8 (2017): e0182738. 28793336 10.1371/journal.pone.0182738 PMC5549998

[40] BTS/NICE/SIGN collaborative guideline NG245 , “Cost‐Utility Analysis: Step‐Up Therapy for Management of Uncontrolled Asthma,” Asthma: Diagnosis, Monitoring and Chronic Asthma Management (Update), (November 2024), https://www.nice.org.uk/guidance/ng245/evidence/costutility-analysis-stepup-therapy-for-management-of-uncontrolled-asthma-pdf-13558289294.

