# Supplementary material for: Cost‐Effectiveness Analysis of Treatments for Children With Uncontrolled Asthma Symptoms Despite Inhaled Corticosteroids
Source: Pediatr Pulmonol. 2025 Dec 15;60(12):e71414. doi: 10.1002/ppul.71414 (PMC12703566; doi:10.1002/ppul.71414)
Supplement: Supplementary file 1 — Supplementary appendix EINSTEIN CEA PP. [file PPUL-60-0-s001.docx]

**Supplementary Appendix**

**EINSTEIN collaborative group**

We thank the following authors for their contribution as part of the EINSTEIN collaborative group: Professors Stanley J. Szefler, Anne M. Fitzpatrick, and David T. Mauger provided IPD and documentation for the INFANT trial via BioLINCC; Professor Chris Frost provided IPD for the publication by Verberne 1998; Professor William D. Carroll provided IPD and documentation for the CHEST trial; Professor Michael E. Wechsler provided IPD and documentation for the BARD trial via BioLINCC; Professor Biju Thomas provided IPD for the ARIDOL trial; Professor Clare S. Murray assisted to retrieve IPD of the GSK trial SAM40100; Professor Robert F. Lemanske provided IPD and documentation for the BADGER trial; Professor Christine A. Sorkness provided IPD and documentation for the PACT trial.

We thank the following companies for their contribution as part of the EINSTEIN collaborative group: GlaxoSmithKline (GSK) Research & Development Ltd (“Trial Sponsor”), 980 Great West Road, Brentford, TW8 9GS, UK | Staffordshire Children's Hospital at Royal Stoke and Keele University, University Hospitals of the North Midlands, Stoke-on-Trent, UK (William D Carroll, MD) | London School of Hygiene and Tropical Medicine, London, UK (Chris Frost, PhD) | Emory University, Department of Pediatrics, Atlanta, GA, USA (Anne M Fitzpatrick, PhD) | Penn State University, College of Medicine, Department of Public Health Sciences, Hershey, PA, USA (David T Mauger, PhD) | University of Colorado, Department of Pediatrics, Anschutz Medical Campus; Children's Hospital Colorado Breathing Institute; University of Colorado Anschutz Medical Campus, Adult and Child Consortium for Outcomes Research and Delivery Science, CO, USA (Stanley J Szefler, MD) | Pediatric Asthma Research Program, The Breathing Institute, Interim Medical Director, Research Institute Children's Hospital Colorado, National Jewish Health and University of Colorado School of Medicine, Denver, CO (Michael E Wechsler, MD, MMSc) | University of Manchester and Manchester University NHS Foundation Trust, UK (Clare S Murray, MBChB, MD, MRCP, MRCPCH) | KK Women's and Children's Hospital and Duke-NUS Medical School, Singapore (Biju Thomas, MBBS, FRCPCH) | University of Wisconsin School of Medicine and Public Health, Madison, WI, USA (Robert F. Lemanske, MD) | School of Pharmacy University of Wisconsin, WI, USA (Christine A Sorkness, PharmD, RPh)

**E-Table 1**. Transition probabilities relating to the weekly rate at which paediatric patients transit amongst health states in the base-case, and the sensitivity where the strength of ICS + LABA is separated.

| Transition to:  Transition from: | Asthma controlled | Asthma uncontrolled | Asthma exacerbation | Death from asthma exacerbation |
| --- | --- | --- | --- | --- |
| Low-dose ICS |  |  |  |  |
| Controlled asthma | 0.93300 | 0.05800 | 0.00900 | 0.00000 |
| Uncontrolled asthma | 0.88700 | 0.09700 | 0.01600 | 0.00000 |
| Asthma exacerbation | 0.25500 | 0.73300 | 0.01200 | 1.92x10^-6^ |
| Medium-dose ICS |  |  |  |  |
| Controlled asthma | 0.93512 | 0.05800 | 0.00688 | 0.00000 |
| Uncontrolled asthma | 0.89561 | 0.09215 | 0.01224 | 0.00000 |
| Asthma exacerbation | 0.25748 | 0.73300 | 0.00952 | 1.5x10^-6^ |
| High-dose ICS |  |  |  |  |
| Controlled asthma | 0.93491 | 0.05800 | 0.00709 | 0.00000 |
| Uncontrolled asthma | 0.81635 | 0.17104 | 0.01261 | 0.00000 |
| Asthma exacerbation | 0.23469 | 0.73300 | 0.03231 | 1.5x10^-6^ |
| Medium-dose ICS + LTRA |  |  |  |  |
| Controlled asthma | 0.93013 | 0.05800 | 0.01187 | 0.00000 |
| Uncontrolled asthma | 0.89101 | 0.08788 | 0.02111 | 0.00000 |
| Asthma exacerbation | 0.25615 | 0.73300 | 0.01084 | 2.5x10^-6^ |
| Death from asthma exacerbation | 0.00000 | 0.00000 | 0.00000 | 1.00000 |
| LTRA |  |  |  |  |
| Controlled asthma | 0.91013 | 0.05800 | 0.03187 | 0.00000 |
| Uncontrolled asthma | 0.50189 | 0.44145 | 0.05666 | 0.00000 |
| Asthma exacerbation | 0.14429 | 0.73300 | 0.12271 | 6.8x10^-6^ |
| ICS + LABA |  |  |  |  |
| Controlled asthma | 0.93474 | 0.05800 | 0.00726 | 0.00000 |
| Uncontrolled asthma | 0.91973 | 0.06737 | 0.01290 | 0.00000 |
| Asthma exacerbation | 0.26441 | 0.73300 | 0.00259 | 1.6x10^-6^ |
| Low-dose ICS + LABA |  |  |  |  |
| Controlled asthma | 0.93465 | 0.05800 | 0.00735 | 0.00000 |
| Uncontrolled asthma | 0.91672 | 0.07021 | 0.01307 | 0.00000 |
| Asthma exacerbation | 0.26355 | 0.73300 | 0.00345 | 1.6x10^-6^ |
| Medium-dose ICS + LABA |  |  |  |  |
| Controlled asthma | 0.93686 | 0.05800 | 0.00514 | 0.00000 |
| Uncontrolled asthma | 0.90422 | 0.08664 | 0.00914 | 0.00000 |
| Asthma exacerbation | 0.25995 | 0.73300 | 0.00705 | 1.1x10^-6^ |
| High-dose ICS + LABA |  |  |  |  |
| Controlled asthma | 0.93305 | 0.05800 | 0.00895 | 0.00000 |
| Uncontrolled asthma | 0.95848 | 0.02560 | 0.01592 | 0.00000 |
| Asthma exacerbation | 0.27000 | 0.71824 | 0.01176 | 1.9x10^-6^ |

**E-Table 2**. Base-case model inputs used in the probabilistic sensitivity analysis

| Parameter | Point estimate | Distribution  (distribution parameters) |
| --- | --- | --- |
| Bayesian predicted probabilities for ‘asthma controlled’ | | |
| Low-dose ICS | 0.7520 | ~ Beta (391.8602, 129.2305) |
| Medium-dose ICS | 0.7690 | ~ Beta (78.9320, 23.7104) |
| High-dose ICS | 0.6867 | ~ Beta (32.5213, 14.8375) |
| ICS + LABA | 0.7798 | ~ Beta (123.6820, 34.9253) |
| ICS + LTRA | 0.7576 | ~ Beta (5.4341, 1.7387) |
| LTRA | 0.4222 | ~ Beta (3.0072, 4.1155) |
| Bayesian predicted probabilities for ‘asthma exacerbation’ | | |
| Low-dose ICS | 0.0594 | ~ Beta (113.7532, 1801.2838) |
| Medium-dose ICS | 0.0551 | ~ Beta (21.2642, 364.6560) |
| High-dose ICS | 0.0439 | ~ Beta (11.7487, 255.8770) |
| ICS + LABA | 0.0479 | ~ Beta (26.3318, 523.3937) |
| ICS + LTRA | 0.0849 | ~ Beta (4.5314, 48.8427) |
| LTRA | 0.2099 | ~ Beta (1.9551, 7.3595) |
| Health state utility (U) | | |
| Asthma controlled | 0.96 | 1-U ~ Gamma (0.0400, 1.0000) |
| Asthma uncontrolled | 0.86 | 1-U ~ Gamma (0.4900, 0.2857) |
| Asthma exacerbation | 0.76 | 1-U ~ Gamma (1.4400, 0.1666) |

**E-Table 3**. Modelled costs and QALYs, disaggregated by health state and treatment.

| Health state  Treatment | Controlled asthma | | Uncontrolled asthma | | Asthma exacerbation | |
| --- | --- | --- | --- | --- | --- | --- |
|  | Costs (£) | QALYs | Costs (£) | QALYs | Costs (£) | QALYs |
| ICS + LABA | 346 | 0.8828 | 38 | 0.0627 | 117 | 0.0058 |
| Medium-dose ICS | 215 | 0.8800 | 28 | 0.0641 | 134 | 0.0067 |
| Low-dose ICS | 119 | 0.8772 | 21 | 0.0660 | 145 | 0.0072 |
| High-dose ICS | 432 | 0.8735 | 51 | 0.0712 | 113 | 0.0055 |
| ICS + LTRA | 232 | 0.8718 | 31 | 0.0673 | 207 | 0.0103 |
| LTRA | 47 | 0.7726 | 31 | 0.1343 | 593 | 0.0297 |

E-Table 4: Results of one-way sensitivity analyses

| Sensitivity analysis | Total cost/(£) | Total QALY | Incremental cost (£) | Incremental QALY | ICER (£/QALY gained) |
| --- | --- | --- | --- | --- | --- |
| Treatment |  |  |  |  |  |
| Cost of high-dose ICS decreased by 50% | | | | | |
| ICS + LABA | 501 | 0.9512 | 124 | 0.0004 | 304,956 |
| Medium-dose ICS | 377 | 0.9508 | 93 | 0.0004 | 255,555 |
| Low-dose ICS | 284 | 0.9504 | – | – | – |
| High-dose ICS | 379 | 0.9503 | – | – | Dominated |
| ICS + LTRA | 470 | 0.9495 | – | – | Dominated |
| LTRA | 670 | 0.9366 | – | – | Dominated |
| Cost of medium-dose ICS decreased by 50% | | | | | |
| ICS + LABA | 501 | 0.9512 | 220 | 0.0004 | 534,712 |
| Medium-dose ICS | 281 | 0.9508 | – | – | – |
| Low-dose ICS | 284 | 0.9504 | – | – | Dominated |
| High-dose ICS | 596 | 0.9503 | – | – | Dominated |
| ICS + LTRA | 470 | 0.9495 | – | – | Dominated |
| LTRA | 670 | 0.9366 | – | – | Dominated |
| Cost of ICS + LABA decreased by 50% | | | | | |
| ICS + LABA | 333 | 0.9512 | 49 | 0.0008 | 63,217 |
| Low-dose ICS | 284 | 0.9504 | – | – | – |
| Medium-dose ICS | 377 | 0.9508 | – | – | Extendedly dominated |
| High-dose ICS | 596 | 0.9503 | – | – | Dominated |
| ICS + LTRA | 470 | 0.9495 | – | – | Dominated |
| LTRA | 670 | 0.9366 | – | – | Dominated |
| Probability of exacerbation increased by 50% | | | | | |
| ICS + LABA | 576 | 0.9430 | 112 | 0.0007 | 161,521 |
| Medium-dose ICS | 464 | 0.9423 | 74 | 0.0050 | 14,797 |
| Low-dose ICS | 390 | 0.9374 | – | – | – |
| High-dose ICS | 671 | 0.9414 | – | – | Dominated |
| ICS + LTRA | 598 | 0.9401 | – | – | Dominated |
| LTRA | 1045 | 0.9190 | – | – | Dominated |
| Utility for uncontrolled asthma increased by 0.05 | | | | | |
| ICS + LABA | 501 | 0.9548 | 217 | 0.0005 | 370,451 |
| Low-dose ICS | 284 | 0.9543 | – | – | – |
| Medium-dose ICS | 377 | 0.9545 | – | – | Extendedly Dominated |
| High-dose ICS | 596 | 0.9544 | – | – | Dominated |
| ICS + LTRA | 470 | 0.9534 | – | – | Dominated |
| LTRA | 670 | 0.9544 | – | – | Dominated |
| Utility for uncontrolled asthma decreased by 0.05 | | | | | |
| ICS + LABA | 501 | 0.9476 | 124 | 0.0005 | 248,800 |
| Medium-dose ICS | 377 | 0.9471 | 93 | 0.0005 | 197,453 |
| Low-dose ICS | 284 | 0.9466 | – | – | – |
| High-dose ICS | 596 | 0.9461 | – | – | Dominated |
| ICS + LTRA | 470 | 0.9455 | – | – | Dominated |
| LTRA | 670 | 0.9288 | – | – | Dominated |
| Utility for asthma exacerbation state increased by 0.05 | | | | | |
| ICS + LABA | 501 | 0.9516 | 124 | 0.0004 | 349,787 |
| Medium-dose ICS | 377 | 0.9512 | 93 | 0.0003 | 284,861 |
| Low-dose ICS | 284 | 0.9509 | – | – | – |
| High-dose ICS | 596 | 0.9506 | – | – | Dominated |
| ICS + LTRA | 470 | 0.9501 | – | – | Dominated |
| LTRA | 670 | 0.9385 | – | – | Dominated |
| Utility for asthma exacerbation state decreased by 0.05 | | | | | |
| ICS + LABA | 501 | 0.9508 | 124 | 0.0005 | 261,241 |
| Medium-dose ICS | 377 | 0.9503 | 93 | 0.0004 | 231,767 |
| Low-dose ICS | 284 | 0.9499 | – | – | – |
| High-dose ICS | 596 | 0.9499 | – | – | Dominated |
| ICS + LTRA | 470 | 0.9488 | – | – | Dominated |
| LTRA | 670 | 0.9346 | – | – | Dominated |
| Utilities taking the values of the lower bound 95% CIs | | | | | |
| ICS + LABA | 501 | 0.9388 | 124 | 0.0004 | 299,812 |
| Medium-dose ICS | 377 | 0.9384 | 93 | 0.0004 | 255,576 |
| Low-dose ICS | 284 | 0.9380 | – | – | – |
| High-dose ICS | 596 | 0.9379 | – | – | Dominated |
| ICS + LTRA | 470 | 0.9371 | – | – | Dominated |
| LTRA | 670 | 0.9242 | – | – | Dominated |
| Utilities taking the values of the upper bound 95% CIs | | | | | |
| ICS + LABA | 501 | 0.9636 | 124 | 0.0004 | 299,670 |
| Medium-dose ICS | 377 | 0.9632 | 93 | 0.0004 | 255,575 |
| Low-dose ICS | 284 | 0.9628 | – | – | – |
| High-dose ICS | 596 | 0.9627 | – | – | Dominated |
| ICS + LTRA | 470 | 0.9619 | – | – | Dominated |
| LTRA | 670 | 0.9490 | – | – | Dominated |
